# Survey on the radiology report at Chris Hani Baragwanath Academic Hospital: Clinician and radiologist perspectives

**DOI:** 10.4102/sajr.v28i1.2954

**Published:** 2024-10-10

**Authors:** Liane Thormahlen, Robyn M. Wessels, Ilana M. Viljoen

**Affiliations:** 1 Division of Diagnostic Radiology, Department of Radiation Sciences, School of Clinical Medicine, Faculty of Health Sciences, University of the Witwatersrand, Johannesburg, South Africa; 2 Division of Radiology, Department of Radiation Medicine, Faculty of Health Sciences, University of Cape Town, Cape Town, South Africa

**Keywords:** survey, radiology, report, clinician, opinion, preferences, utilisation, clinical history

## Abstract

**Background:**

The radiology report is the primary means of conveying imaging findings between radiologists and clinicians. As a result, clinician satisfaction with the radiology report is an indicator of its quality and clinical relevance. It is crucial to identify factors that can enhance the radiology report in order to improve service delivery.

**Objectives:**

This study evaluates clinician and radiologist opinions, preferences and clinician utilisation of the radiology report.

**Method:**

Mixed quantitative and qualitative survey questionnaires were distributed in-person and online from December 2022 to February 2023 to a total of 287 clinicians and 43 independent medical practitioners specialising in radiology.

**Results:**

A total of 73.0% of radiologists and 56.5% of clinicians expressed satisfaction with the radiology reports. Additionally, 72.0% of radiologists expressed dissatisfaction with the history provided on the referral forms. It was found that 87.6% of clinicians read the radiology report, while 26.2% reviewed the radiological imaging without referring to it. Interestingly, 77.8% of clinicians preferred itemised listed reports, whereas 53.8% of radiologists preferred reports in paragraph format. It was discovered that 69.6% of radiologists and 65.4% of clinicians preferred a standardised reporting format.

**Conclusion:**

More than half of the clinicians and most of the radiologists expressed satisfaction with the radiology report. Both clinicians and radiologists showed a preference for a structured reporting format. A crucial element in constructing a good radiology report was having a relevant clinical history. The radiologist continued to be the preferred professional for interpreting radiological imaging.

**Contribution:**

This survey was a good starting point for improving communication between clinicians and radiologists. This will ultimately result in reports that are more useful to clinicians and radiologists who have a better understanding of what should be included in reports and how they should be structured.

## Introduction

The radiology report is the primary method of communicating findings of imaging studies and is therefore the main product of service delivery in a radiological department.^1^ It is a medicolegal document and the official interpretation of single or multiple radiological examinations. Although other forms of digital and in-person communication do exist, such as face-to-face meetings, multidisciplinary meetings and direct telephone communication, in resource-limited settings, the radiology report, at times, remains the only tool of communication between radiologists and clinicians.^1,2,3,4^

The style and content of the radiology report can differ vastly depending on the reporting radiologist resulting in a variable layout, style and length. The ideal radiology report remains an ongoing, contentious debate within the discipline.^2,5^

Several international studies have analysed the perception, satisfaction and preferred structure and content of the radiology report.^4,5,6,7,8,9,10,11,12^ A study conducted in the Netherlands and Flanders found that the majority of referring clinicians were satisfied with the content of the radiology reports.^6,7^ Overall, an itemised reporting structure was preferred.^5,10,11^

When radiologists were asked to reflect on the quality and structure of their own reports, most believed their reports to be of high quality regardless of the structure.^6^ Personal preferences with regard to style, the need for standardisation of reports, and the use of lexicons are varied among the disciplines.^12,13,14,15^

In Africa, a limited number of studies analysing the radiology report have been conducted. One such study from Kenya, highlighted the challenges faced in a resource-limited setting. The biggest hurdle faced by these radiologists was the unavailability of the Reporting Information System (RIS) and Picture Archiving and Communication System (PACS) resulting in handwritten and manually delivered radiology reports. This in turn resulted in a delay in communication of pertinent findings especially when no contact details were provided by clinicians on referral forms.^4^

To date, no other studies analysing the radiology report have been conducted in Africa which prevents any viable conclusions from being drawn on the local experience. The paucity of literature on this topic in South Africa formed the rationale for conducting this research report with the aim to evaluate clinician and radiologist opinions, preferences and utilisation of the radiology report at Chris Hani Baragwanath Academic Hospital (CHBAH).

## Research methods and design

Permission to conduct both surveys at CHBAH was obtained from the head of the department of Diagnostic Radiology and the hospital’s Chief Executive Officer. Chris Hani Baragwanath Academic Hospital is a tertiary level 3400-bed hospital in Johannesburg, Gauteng, South Africa, and the largest hospital in Africa.^16^

A prospective, mixed quantitative and qualitative survey was performed. The clinician and radiologist survey questionnaires were adapted from the COVER/ROVER study conducted by Bosman et al. in the Netherlands and Flanders.^6^ A three-part clinician perspective questionnaire consisting of 31 questions, and a four-part radiology perspective questionnaire consisting of 25 questions, were developed.

In the first section of both questionnaires, participants were asked to enter demographic data. The second and third parts of the clinician questionnaire assessed the opinion, preferences and utilisation of the radiology report (twenty-four statements) and clinician interpretation of imaging (six statements), respectively. The second, third and fourth parts of the radiologist questionnaires assessed the opinion on the radiology report (eight statements), clinical history (four statements) and structure of the radiology report (ten statements), respectively. All the statements in the second and third parts of the clinician questionnaire and the second to fourth parts of the radiology questionnaire were assessed using the five-part Likert scale.

Clinicians and radiologists make use of a PACS where all radiology imaging and typed reports are available. In addition to the radiology department, all emergency units, intensive care units, most wards and some theatres have ready access to the PACS. No radiology imaging or reports at CHBAH are accessible via mobile devices.

For the data collection, both the clinician and radiologists’ questionnaires were distributed in a mixed format, during individual and joint departmental meetings in a classroom setup as well as by online links that were sent via the official university platform. All clinicians registered as independent medical practitioners or specialists with the Health Professions Council of South Africa (HPCSA) (medical officers, registrars, consultants and fellows, including heads of units and departments), that rotated through CHBAH, and utilised the services of the radiology department during the data collection period were included in the study. All medical officers, registrars and consultants working in the Department of Radiology at CHBAH at the time of data collection were included in the study.

A clinician response rate of more than 40.0% was expected for the questionnaires distributed in a classroom setup and 20.0% – 30.0% response rate was expected for the online surveys. It was expected that 50.0% – 60.0% of radiologists would complete the questionnaire. The time allowed for data collection was approximately 3 months (13 weeks). Both groups received reminders to complete their respective surveys twice a month at interdisciplinary meetings with radiology, in person during their departmental meetings and via email communication.

The data obtained from the questionnaires were exported into Excel and all incomplete surveys were excluded from the study. An incomplete survey was defined as one where less than 50% of the questions were completed. Statistical analysis was conducted using IBM SPSS Statistics version 28. The Likert scale was analysed by combining the results into three categories: agree (strongly agree and agree), neutral and disagree (disagree and strongly disagree). Descriptive statistics were presented in frequencies and percentages for categorical variables and as means with standard deviation for continuous variables. The Pearson Chi-square and Fisher’s exact tests were used to test for correlations between categorical variables, while the independent t-test and one-way analysis of variance (ANOVA) were used to compare the mean outcomes between groups. Pearson correlation coefficient tested for linear relationships between continuous variables. Significance testing was set at the 95% confidence level and a *p*-value less than 0.05 indicated statistical significance.

### Ethical considerations

The study was approved by the Human Research Ethics Committee of the University of the Witwatersrand (reference number M220639). The participants each received a participant information sheet regarding the study, and participation was entirely voluntary. Written informed consent was obtained from all participants. All surveys were completed anonymously, and stored securely to ensure protection of the obtained data.

## Results

All questionnaires were assessed for completeness. No surveys met the criteria for exclusion and were thus all included in the data analysis.

**FIGURE 1:**
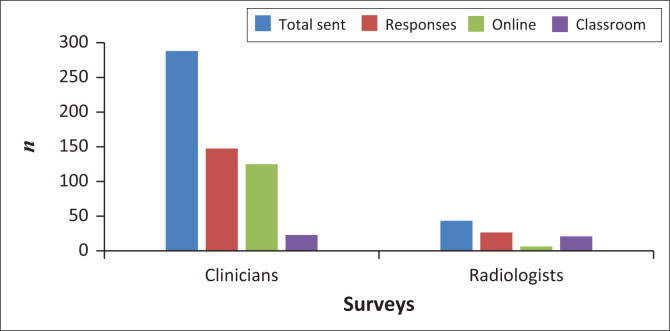
Questionnaire response rates.

Of the distributed 287 clinician questionnaires, 146 clinicians completed the questionnaire. A response rate of 85.0% was achieved with the online link and a 15.0% response rate was achieved from those questionnaires distributed in a classroom setup. A total clinician response rate of 50.9% was achieved (Figure 1). Forty-three questionnaires were distributed to radiologists, of which 26 were completed. A response rate of 76.9% was reached in the classroom setup and 23.1% with the online links. A total radiology response rate of 60.0% was achieved (Figure 1). An overall response rate of 55.4% was achieved for both surveys.

**FIGURE 2:**
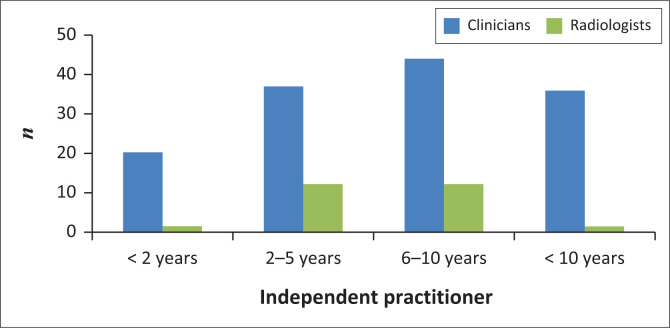
Years registered as an independent medical practitioner.

Within the clinician group, 53.4% were male. The highest clinician response rate was achieved from the internal medicine department at 44.5% (Table 1). Most respondents were registrars (44.1%), followed by consultants (32.2%) and medical officers (21.7%). Registrar responses were distributed almost equally among first to fourth years. Participating consultants demonstrated a wide range of experience, the majority (40%) of whom had more than 10 years of experience. Most participating clinicians have been independent medical practitioners for 6–10 years (Figure 2). The majority (53.9%) of the participating clinicians had not worked in another department prior to joining their current department. Of those who have worked in other departments, most spent time in the intensive care unit (18.5%).

**TABLE 1:** Clinician response rate per department.

Department	Frequency	%
**Clinician response rate per department**		
Accident and emergency	15	10.3
Anaesthetics	5	3.4
Dermatology	2	1.4
Family medicine	3	2.1
Intensive care unit	4	2.8
Internal medical	65	44.5
Nuclear medicine	1	0.7
Obstetrics and gynaecology	5	3.4
Paediatrics	4	2.7
Psychiatry	1	0.7
Surgical (including paediatric surgery)	40	27.4
Radiation oncology	1	0.7

**Total**	**146**	**100.0**

In contrast to the participants from the clinician survey, 61.5% of the radiologists participating in the survey were female. Most participants in the radiology department were registrars (69.2%). Of the registrars who participated in the study, 55.6% were first and second year in a 4-year training programme. Sixty per cent of the participating consultants had less than 2 years’ experience as radiology consultants. The participating radiologists have been independent medical practitioners between 2 and 10 years (Figure 2). The majority (73.1%) of radiologists previously worked in clinical departments, most having worked in the accident and emergency department (36.8%).

### Opinion on the radiology report

#### Satisfaction with the radiology report

Just over half (56.5%) of clinicians were satisfied with the radiology report at CHBAH. Most radiologists (73.0%) were satisfied with their own reports (Table 2).

**TABLE 2:** Opinion on the radiology report.

Opinion on the radiology report	Strongly disagree		Disagree		Disagree (total)		Neutral		Agree		Strongly agree		Agree (total)		Total	
	*n*	%	*n*	%	*n*	%	*n*	%	*n*	%	*n*	%	*n*	%	*N*	%
**Clinicians**																
The quality of radiology reports at CHBAH is satisfactory.	4	2.8	16	11.0	20	13.8	43	29.7	73	50.3	9	6.2	82	56.6	145	100.0
The radiology report always answers the clinical question.	4	2.8	57	39.6	61	42.4	42	29.2	35	24.3	6	4.2	41	28.5	144	100.0
The radiology report highlights aspects I did not clinically appreciate myself.	2	1.4	13	9.1	15	10.5	31	21.7	74	51.7	23	16.1	97	67.8	143	100.0
I prefer it when a comparison is made between current and previous imaging in the radiology report.	3	2.1	1	0.7	4	2.8	3	2.1	16	11.0	122	84.1	138	95.2	145	100.0
I prefer it when the radiology report recommends further radiological imaging (if appropriate).	2	1.4	1	0.7	3	2.1	11	7.5	28	19.2	104	71.2	132	90.4	146	100.0
I prefer it when the radiology report recommends a further management plan (for example: Serology, advice on further workup, referral to another department, etc.).	19	13.0	14	9.6	33	22.6	40	27.4	29	19.9	44	30.1	73	50.0	146	100.0
The quality of the radiology report is directly proportional to the amount of the clinical history provided.	5	3.4	16	11.0	21	14.5	44	30.3	55	37.9	25	17.2	80	55.2	145	100.0
Providing a pertinent clinical question improves the quality of the radiology report.	2	1.4	9	6.3	11	7.6	23	16.0	57	39.6	53	36.8	110	76.4	144	100.0
**Radiologists**																
I am generally happy with the quality of my radiology reports.	1	3.8	0	0.0	1	3.8	6	23.1	16	61.5	3	11.5	19	73.1	26	100.0
If there is a clinical question on the clinician referral form, I answer it in my radiology report.	1	3.8	0	0.0	1	3.8	1	3.8	9	34.6	15	57.7	24	92.3	26	100.0
The radiology report identifies pathology NOT appreciated on initial patient assessment by clinicians.	0	0.0	1	3.8	1	3.8	4	15.4	10	38.5	11	42.3	21	80.8	26	100.0
When I report the current requested imaging, I always compare it to previous imaging (if available).	1	4.2	0	0.0	1	4.2	0	0.0	3	12.5	20	83.3	23	95.8	24	100.0
When reviewing radiological imaging, it is important to recommend further imaging if required.	1	3.8	0	0.0	1	3.8	2	7.7	9	34.6	14	53.8	23	88.5	26	100.0
When reviewing radiological imaging, it is important to recommend a further management plan if required.	1	3.8	1	3.8	2	7.7	4	15.4	11	42.3	9	34.6	20	76.9	26	100.0
I can construct a good radiology report regardless of the adequacy of the clinical history.	2	7.7	8	30.8	10	38.5	6	23.1	10	38.5	0	0.0	10	38.5	26	100.0
There should always be a clinical question on the radiology request form (in addition to the clinical history).	1	3.8	0	0.0	1	3.8	2	7.7	5	19.2	18	69.2	23	88.5	26	100.0
I can construct a good radiology report regardless of whether a clinical question has been asked.	0	0.0	2	7.7	2	7.7	12	46.2	10	38.5	2	7.7	12	46.2	26	100.0
I am satisfied with the patient history provided by clinicians at CHBAH on radiology request forms.	7	28.0	10	40.0	17	68.0	8	32.0	0	0.0	0	0.0	0	0.0	25	100.0

CHBAH, Chris Hani Baragwanath Academic Hospital.

#### Does the report answer the clinical question?

Most clinicians (42.4%) felt that the radiology report did not answer the clinical question. Many clinicians remained neutral (29.2%) and the minority (28.5%) were in agreement that the radiology report answered the clinical question. Contrary to this, 92.3% of the participating radiologists agreed that they answered the clinical question if it was available on the clinician referral form.

#### Does the radiology report add value to the clinical examination?

More than two-thirds (67.8%) of clinicians and 80.8% of radiologists agreed that the radiology report highlighted aspects that were not appreciated at the time of the clinical examination.

#### How much should the radiologist recommend in the radiology report?

A total of 88.4% of radiologists agreed that it is important to recommend further appropriate imaging in their reports which was echoed by 91.4% of clinicians. The importance of recommending a further management plan was agreed upon by 76.9% of radiologists and half of the clinicians.

#### Does providing an adequate history or pertinent clinical question improve the quality of the radiology report?

In response to whether they felt that the quality of the radiology report was directly proportional to the provided clinical history, just over half (55.1%) of the clinicians agreed. A total of 64.0% of clinicians agreed that providing a pertinent clinical question to the reporting radiologist would improve the radiology report. Additionally, 88.4% of radiologists agreed that a pertinent question, in addition to the clinical history, should always be on clinician referrals. However, 72.0% of participating radiologists were dissatisfied with the history of clinician referral forms.

When the reporting radiologists were asked whether they could construct a good report without a clinical history, the results were equivocal. A total of 38.5% responded that they could still construct a good radiology report regardless of the adequacy of the clinical history, but the same percentage responded that they could not. However, 46.2% of reporting radiologists felt that they could construct a good report in the absence of a pertinent clinical question. Regardless of the quality and availability of clinician history and a pertinent clinical question, 95.8% of radiologists responded that they compare current to previous imaging if available.

### Radiology report preferences

#### How much information should the radiology report contain?

A total of 77.8% of clinicians indicated that they would like the reporting radiologist to address all individual organ systems, whether they are normal or not (Table 3). Furthermore, 91.7% of clinicians expressed a preference for a final comment in addition to the imaging findings mentioned in the body of the report. Similarly, 89.6% of clinicians preferred that the radiologist provide a diagnosis in the final comment.

**TABLE 3:** Preferences of the radiology report.

Preferences of the radiology report	Strongly disagree		Disagree		Disagree (total)		Neutral		Agree		Strongly agree		Agree (total)		Total	
	*n*	%	*n*	%	*n*	%	*n*	%	*n*	%	*n*	%	*n*	%	*N*	%
**Clinicians**																
**General preferences**																
I prefer the radiology report to address all the individual organ systems whether they are normal or not.	1	0.7	12	8.3	13	9.0	19	13.2	33	22.9	79	54.9	112	77.8	144	100.0
I prefer it when the reporting radiologist mentions the imaging findings only with no final comment.	106	73.1	27	18.6	133	91.7	4	2.8	5	3.4	3	2.1	8	5.5	145	100.0
I prefer it when the reporting radiologist makes a diagnosis in the final comment.	2	1.4	4	2.8	6	4.2	9	6.3	51	35.4	78	54.2	129	89.6	144	100.0
**Structural preferences**																
I prefer the body of the radiology report to be in paragraph form.	39	26.9	45	31.0	84	57.9	43	29.7	10	6.9	8	5.5	18	12.4	145	100.0
I prefer the body of the radiology report to be in an itemised or bullet point form.	3	2.1	3	2.1	6	4.2	32	22.2	40	27.8	66	45.8	106	73.6	144	100.0
I prefer the radiology report to be written in a standard, predetermined reporting format that is used by all radiologists in the institution.	1	0.7	3	2.1	4	2.8	40	27.6	37	25.5	64	44.1	101	69.7	145	100.0
**Radiologists**																
**Structural preferences**																
I prefer to convey findings in paragraphs.	5	19.2	9	34.6	14	53.8	5	19.2	5	19.2	2	7.7	7	26.9	26	100.0
I prefer to convey findings in bullet point format.	1	3.8	4	15.4	5	19.2	4	15.4	9	34.6	8	30.8	17	65.4	26	100.0
I would prefer to report using a standardised reporting format used by all radiologists in my institution.	0	0.0	5	19.2	5	19.2	4	15.4	7	26.9	10	38.5	17	65.4	26	100.0
I have received formal lectures on how to structure my radiological report.	13	52.0	8	32.0	21	84.0	1	4.0	1	4.0	2	8.0	3	12.0	25	100.0
I learnt to structure my radiological reports by following the reporting templates available in the department.	2	7.7	1	3.8	3	11.5	2	7.7	5	19.2	16	61.5	21	80.8	26	100.0
I learnt to structure my radiological reports by reviewing the reports of my colleagues (registrars and/or consultants).	1	3.8	2	7.7	3	11.5	5	19.2	13	50.0	5	19.2	18	69.2	26	100.0
I would like formal training on the structuring of radiological reports.	0	0.0	2	7.7	2	7.7	7	26.9	4	15.4	13	50.0	17	65.4	26	100.0

#### What should the radiology report look like?

In terms of the body of radiology report, clinicians preferred itemised (77.8%) over paragraphed (12.3%) reports. A total of 65.4% of participating radiologists agreed with the clinician’s preference for itemised reporting; however, just over half (53.8%) of the participating radiologists admitted to also reporting in paragraphs.

#### How does the reporting radiologist decide on report structure?

A total of 88.9% of radiology registrars and 40.0% of consultants use departmental templates created by senior staff members for their reports. The remaining professionals prefer to personalise their reporting structure. It is important to note that the reporting templates are similar, but they are not standardised across all hospitals in the training circuit, and their use is not mandatory. Furthermore, 77.7% of radiology registrars learn about reporting structure by reviewing reports from consultants and fellow registrars. Registrars receive informal training on report structure by analysing reports that have been assessed by consultants, as well as through in-person discussions with radiology consultants while reviewing imaging. Interestingly, 83.3% of radiology registrars and 80.0% of consultants have not received formal lectures on structuring radiology reports. Among the registrars, 77.8% expressed a desire for formal training on report structure. Among the consultants, 80.0% have a neutral stance, while the remaining 20.0% do not wish to receive formal lectures on reporting structure. When both clinicians and radiologists were asked whether they would prefer a standardised institutional reporting format, 69.6% of radiologists and 65.4% of clinicians agreed that they would (Table 3).

### Utilisation of the radiology report

#### Do clinicians use the radiology report?

A total of 87.6% of clinicians read the radiology report regardless of whether it is the provisional registrar or finalised consultant-reviewed report (Table 4). Just over half (51.4%) of clinicians read the radiology report the moment it is available, which are usually provisional registrar reports. This is especially the case after hours, where provisional registrar reports from the previous night are only reviewed by a consultant the following morning and are consequently subject to change. In addition, 72.0% of clinicians stated that they read the entire body of the report. In a separate question, 67.1% of clinicians stated that they only read the comment at the end of the report.

**TABLE 4:** Utilisation of the radiology report.

General preferences	Strongly disagree		Disagree		Disagree (total)		Neutral		Agree		Strongly agree		Agree (total)		Total	
	*n*	%	*n*	%	*n*	%	*n*	%	*n*	%	*n*	%	*n*	%	*N*	%
**Clinicians**																
I read the radiology report the moment it was available.	4	2.8	22	15.2	26	17.9	43	29.7	30	20.7	46	31.7	76	52.4	145	100.0
I read the entire radiology report.	4	2.7	16	11.0	20	13.7	21	14.4	36	24.7	69	47.3	105	71.9	146	100.0
I only read the comments at the end of the radiology report.	64	43.8	34	23.3	98	67.1	19	13.0	20	13.7	9	6.2	29	19.9	146	100.0
I do not read the radiology report.	108	74.5	19	13.1	127	87.6	6	4.1	9	6.2	3	2.1	12	8.3	145	100.0
I only read the provisional radiology report.	34	23.4	47	32.4	81	55.9	43	29.7	17	11.7	4	2.8	21	14.5	145	100.0
I only read the final radiology report.	21	14.4	46	31.5	67	45.9	45	30.8	23	15.8	11	7.5	34	23.3	146	100.0
**Radiologists**																
The clinicians look at the body of the radiology report in addition to the comment section.	0	0.0	16	61.5	16	61.5	6	23.1	2	7.7	2	7.7	4	15.4	26	100.0
The clinicians only look at the comment section at the end of the radiology report.	1	3.8	0	0.0	1	3.8	3	11.5	12	46.2	10	38.5	22	84.6	26	100.0
I would like formal training on the structuring of radiological reports.	0	0.0	2	7.7	2	7.7	7	26.9	4	15.4	13	50.0	17	65.4	26	100.0

When radiologists were asked how they thought clinicians utilised their reports, 84.7% believed clinicians only looked at the comment section at the end of the radiology report. Additionally, 61.5% of these radiologists believed clinicians did not look at the body of their reports. The minority (15.4%) of radiologists thought that clinicians read their entire radiology report.

### Improvements to the radiology report

#### Is there something specific that would make the radiology report more relevant to the clinician?

Of the 49.3% of clinicians who participated in the open-ended response question, 11 (8.0%) of clinicians emphasised addressing the clinical question in the final comment. Five (3.0%) of clinicians wanted the radiologist to include more differential diagnoses if appropriate and ensure greater diagnostic accuracy. Three (2.0%) of clinicians felt that a standardised reporting structure would improve the radiology report. Timeous consultant review of provisional reports and timeous performance of requested imaging were cited by seven (5.0%) of clinicians, respectively.

### Image interpretation and training

#### Do clinicians interpret radiological imaging?

A total of 79.4% of clinicians review radiological imaging when they have access to it; however, only 26.2% of clinicians prefer to review imaging without reading the radiology report (Table 5). The highest frequency of clinician-reviewed cases per week was 11–20. This contrasts with an average of 25–50 radiologist-reviewed cases per week. Clinicians who have been independent medical practitioners for longer, reviewed more cases without a formal radiology report per week (*p* < 0.05). X-rays (95.4%) and CT (81.5%) were the most self-interpreted modalities. Less experienced clinicians relied more heavily on the findings of the radiology report. Clinicians felt most confident in their interpretation of X-rays (88.2%) and CT (58.4%) in comparison to ultrasound (27.5%), MRI (23.4%) and fluoroscopy (10.4%).

**TABLE 5:** Image interpretation and training.

Clinician image interpretation and training	Strongly disagree		Disagree		Disagree (total)		Neutral		Agree		Strongly agree		Agree (total)		Total	
	*n*	%	*n*	%	*n*	%	*n*	%	*n*	%	*n*	%	*n*	%	*N*	%
When I can, I review radiological imaging myself (printed films or on PACS).	4	2.7	10	6.8	14	9.6	16	11.0	45	30.8	71	48.6	116	79.5	146	100.0
I would prefer to review radiological imaging myself without reading the radiology report (if I had ready access to radiological imaging).	45	31.0	34	23.4	79	54.5	28	19.3	16	11.0	22	15.2	38	26.2	145	100.0
**Confidence in image interpretation**																
I feel confident in my interpretation of X-rays.	2	1.4	0	0.0	2	1.4	15	10.4	74	51.4	53	36.8	127	88.2	144	100.0
I feel confident in my interpretation of CT.	6	4.2	17	12.0	23	16.2	36	25.4	50	35.2	33	23.2	83	58.5	142	100.0
I feel confident in my interpretation of Ultrasound.	20	13.9	36	25.0	56	38.9	34	23.6	36	25.0	18	12.5	54	37.5	144	100.0
I feel confident in my interpretation of MRI.	53	36.6	31	21.4	84	57.9	27	18.6	25	17.2	9	6.2	34	23.4	145	100.0
I feel confident in my interpretation of Fluoroscopy.	81	56.3	24	16.7	105	72.9	24	16.7	11	7.6	4	2.8	15	10.4	144	100.0
**Image interpretation training**																
I have had training (informal and formal) in the interpretation of radiological imaging.	6	4.3	12	8.5	18	12.8	22	15.6	45	31.9	56	39.7	101	71.6	141	100.0
I have had training in the interpretation of X-rays.	2	1.4	3	2.1	5	3.5	10	6.9	61	42.4	68	47.2	129	89.6	144	100.0
I have had training in the interpretation of CT.	6	4.2	15	10.5	21	14.7	29	20.3	50	35.0	43	30.1	93	65.0	143	100.0
I have had training in the interpretation of Ultrasound.	21	14.7	27	18.9	48	33.6	26	18.2	37	25.9	32	22.4	69	48.3	143	100.0
I have had training in the interpretation of MRI.	55	38.2	29	20.1	84	58.3	24	16.7	23	16.0	13	9.0	36	25.0	144	100.0
I have had training in the interpretation of Fluoroscopy.	83	57.6	29	20.1	112	77.8	12	8.3	10	6.9	10	6.9	20	13.9	144	100.0
I would benefit from dedicated radiological imaging interpretation training.	3	2.1	3	2.1	6	4.1	23	15.9	29	20.0	84	57.9	113	77.9	145	100.0
I do not think that dedicated radiological imaging interpretation training is necessary as a clinician.	102	70.3	19	13.1	121	83.4	9	6.2	10	6.9	5	3.4	15	10.3	145	100.0

PACS, picture archiving and communication system; CT, computed tomography; MRI, magnetic resonance imaging.

All radiologists reported CT and fluoroscopy, and 92.3% reported X-rays, 80.8% ultrasound, 73.1% MRI, and only 11.5% mammography. Among the radiologists, CT was the most interpreted modality per week (96.2%).

#### Do you receive formal or informal training in image interpretation as a clinician?

A total of 71.6% of clinicians have had training, informal and formal, in radiological image interpretation, predominantly in X-rays (89.6%) and CT (65.1%). Most clinicians (77.9%) agreed that further imaging interpretation training would be beneficial. Additionally, 83.4% also agreed that formal and informal radiological training is a necessary part of clinician speciality training.

## Discussion

There appears to be a disconnect between clinician expectations, and the perception of radiologists as to what constitutes a good report. Failure to answer the clinical question was cited as a cause for clinician dissatisfaction; however, this was largely disputed among the radiologists. Over half of clinicians thought that an adequate clinical history (55.5%) and pertinent clinical questions (64.0%) were fundamental to a good radiology report which is in line with multiple international surveys.^6,7,17,18,19^ In contradistinction, almost three-quarters of radiologists were dissatisfied with the quality of the clinical history (72.9%) and pertinent clinical questions (88.4%) on referral forms which echoed the sentiments of their international colleagues.^6^ Whether radiologists could still construct a good radiology report without an adequate clinical history and pertinent clinical questions remained equivocal.

The structural preferences of the radiology report have been assessed internationally with clinicians preferring itemised over paragraphed reports.^3,4,5,6,9^ However, the report structure is determined by individual preferences among radiologists with significant inter-report variation and no standardisation. Like CHBAH clinicians, clinicians in surveys by Bosmans et al. and Wairimu et al. found itemised and structured reports easier to read and recall.^4,6^ Additionally, they were more often used in patient management in comparison to paragraph reports.^4,5,6,8,9,10,11,12^ Approximately two-thirds of CHBAH clinicians (65.4%) and radiologists (69.6%) would prefer the radiology report to be standardised.

Radiologists believed that formal training on reporting structure would improve and contribute to the standardisation of the radiology report. Formal training of registrars may be introduced into the existing curriculum by including an approach to standardised reporting and sample templates in topic-based lectures across the training circuit.

Despite the perceived shortcomings of the radiology report, 87.6% of clinicians at CHBAH still use it, with over half reading it as soon as it is available. Both clinicians and radiologists believe that the radiology report adds value to patient management. The amount of information included in reports varied among radiologists, while most clinicians preferred to have as much information as possible.^4,6,7,9^ At CHBAH, 77.8% of clinicians prefer the radiologist to address all the individual organ systems, normal or not, with 91.7% desiring an additional final comment in the report. However, just over two-thirds of clinicians only read the comment section of the report. This raises the question of whether the lack of a standardised reporting structure, particularly in the report body, contributes to the clinician only reading the final comment.

Another debated subject among clinicians and radiologists is how far the reporting radiologists should go in their attempt to contribute to patient management.^4,6,7^ Most clinicians expect the reporting radiologist to make a diagnosis in addition to their final comment. A total of 88.4% of radiologists believed that they should recommend further appropriate imaging and 76.9% of radiologists suggested a management plan in their final comment. Less than a third of clinicians (30.1%) preferred the radiologist to recommend a further management plan. It is thus important to consider whether recommending further management for patients is the responsibility of the radiologist who only has the imaging findings to correlate with the provided clinician-dependant history. Additionally, in a training institution like CHBAH, the imaging request form is most often completed by junior doctors (interns) who do not always covey the main clinical concern, thus making it challenging for the radiologist to suggest a management plan. To construct a thorough, holistic radiology report, 95.8% of radiologists reported that they ensured they reviewed comparative imaging.

Even though clinicians value the radiology report, 79.4% of clinicians still elected to interpret radiological imaging themselves when they can, but only the minority preferred to review imaging without a radiology report. Experienced clinicians were more confident in their review of cases without a formal radiology report in comparison to their colleagues with less experience (*p* < 0.05). X-rays and CT were the most interpreted modalities. Overall, radiologist image interpretation was still preferred, which is in line with findings of a survey in the Netherlands and Flanders.^6^ A total of 77.9% of CHBAH clinicians expressed a desire for training in radiological image interpretation and 83.4% thought it a necessity, thus highlighting the limitations of imaging review by clinicians. Of interest in this survey is that an equal percentage of radiologists lacked formal training on reporting structure as clinicians who had had training in the interpretation of radiological imaging. This emphasises that dedicated training in image interpretation and reporting structure would be beneficial to both the clinician and radiologist, respectively.

The question of how a radiologist learns to report becomes a necessary area of investigation. In keeping with the international literature, little time is spent on teaching radiology registrars how to structure a report.^4,6,7^ Similarly, the majority of CHBAH registrars learn to structure their reports by using departmental templates as guidelines or via review of fellow registrar and consultant reports. Most of the responding registrars did not receive, but expressed a desire for formal lectures on reporting structure. One may infer that training radiologists on reporting structure will improve service delivery as the radiology report is the main tool of communication with clinicians.

### Limitations and recommendations

The survey, although a mixed quantitative and qualitative study, collected subjective data from clinicians and radiologists at a single academic centre. Because of the diverse nature of health care settings in South Africa, findings may differ in settings where there are either more senior or more junior doctors answering the survey questions. Thus, provincial, or national perspectives cannot be inferred. The response rate was higher than in similar international studies; however, a larger sample size of clinicians and radiologists would offer greater insight. The nonresponse bias remains a factor in this study in that the views of responders and non-responders might differ.

## Conclusion

Given the exponential increase in radiological imaging requests, it becomes important to discern what a good radiology report should include, and whether the current reporting structure conveys the radiologists’ findings effectively. There is a disconnect between clinician expectations of the radiology report, and what radiologists perceive as a good report. This survey is a starting point for improving communication between radiologists and clinicians. Standardising the report structure could decrease inter-reporter variability among radiologists which could make the radiology report more accessible to clinicians of all levels of experience. Educating clinicians on what constitutes a good clinical history will aid radiologists in providing a more accurate report with a narrower differential and more precise diagnosis. Providing formal training in image interpretation to clinicians and reporting structure to radiologists could result in clinicians with a greater understanding of imaging pertaining to their speciality, more useful reports from the radiologists and effectively, better communication between the two disciplines. In the future, more detailed surveys and interim reviews could be conducted to monitor the efficacy of the radiology report and guide future adjustments to the departmental reporting structure.
